# Application of machine learning to identify risk factors for bloodstream infections caused by different antimicrobial resistance phenotypes in chemotherapy patients

**DOI:** 10.1007/s00520-026-11379-2

**Published:** 2026-10-20

**Authors:** Adhemar Villani Junior, Maristela P. Freire, Luis Alberto De Padua Covas Lage, Felippe Lazar Neto, Maura Salaroli Oliveira, Edson Abdala, Fatima L. S. Nunes, Anna Sara S. Levin

**Affiliations:** 1 School of Arts, Sciences and Humanities, University of Sao Paulo, Sao Paulo, Brazil; 2 Infectious Diseases Service, Cancer Institute, University of São Paulo School of Medicine, Sao Paulo, Brazil; 3 Department of Infectious Diseases, University of São Paulo School of Medicine, Dr Eneas de Carvalho Aguiar Street, 255, Sao Paulo, SP 05403-900 Brazil; 4 Department of Hematology, Cancer Institute, University of Sao Paulo School of Medicine, Sao Paulo, Brazil; 5 Department of Oncology, Cancer Institute, University of Sao Paulo School of Medicine, Sao Paulo, Brazil; 6 CAMO-Net - Centre for Antimicrobial Optimisation Network, London, UK

**Keywords:** Carbapenem-resistant Gram-negative, Multi-label machine learning classifiers, Antitumor antibiotics, Multidrug resistance, Enterobacterales

## Abstract

**Purpose:**

Bloodstream infections (BSIs) remain a major cause of morbidity and mortality in patients undergoing chemotherapy. The rising incidence of multidrug-resistant organisms (MDROs) further challenges the adequacy of empirical antimicrobial therapy. Therefore, this study aimed to identify features associated with different microbiological profiles of BSIs in chemotherapy patients using machine learning models.

**Methods:**

We retrospectively analyzed 1435 confirmed BSI episodes among 1220 patients treated at a tertiary cancer center from 2017 to 2022. A multi-label classification approach was used to model BSIs associated with carbapenem-resistant Gram-negative bacilli (CR-GNB), methicillin-resistant staphylococci, third-generation cephalosporin-resistant Enterobacterales (3GCRE), fungal pathogens, and non-MDRO/non-fungal. Classifier chain models based on logistic regression, random forests, neural networks, and gradient boosting were trained and evaluated using cross-validation with an independent test set. Model outputs were interpreted using SHapley Additive exPlanations (SHAP) to estimate feature contributions.

**Results:**

Most BSIs occurred during the first chemotherapy cycle and were due to non-MDROs or fungal agents (1193 episodes; 83.1%), CR-GNB was identified in 109 (7.6%). Model performance varied across pathogen groups, with the highest discrimination observed for CR-GNB (AUC-ROC 83.25%; 95% CI 73.91%–91.64%). Key features included hospitalization days, hematologic malignancy, prior MDRO infection, monocytopenia, and palliative chemotherapy. Chemotherapy class contributed differently across pathogen groups, with alkylating agents contributing to candidemia, antitumor antibiotics to CR-GNB, and antimetabolites to 3GCRE. Prior CR-GNB infection was the strongest predictor for CR-GNB BSI, while palliative chemotherapy correlated with non-MDRO/non-fungal BSI.

**Conclusion:**

Multi-label machine learning approaches may help disentangle the complex etiologies of BSIs in cancer patients by identifying associations between host factors, prior MDRO infection, and chemotherapy class within confirmed BSI episodes, informing future hypothesis generation.

**Supplementary information:**

The online version contains supplementary material available at https://doi.org/10.1007/s00520-026-11379-2.

## Introduction

Infections are one of the most frequent causes of morbidity and mortality in patients undergoing chemotherapy. Despite guideline-based recommendations for empirical antimicrobial therapy in febrile neutropenia (FN), including indications for Gram-positive coverage, the rate of inappropriate initial treatment remains substantial [1]. In a Spanish cohort of patients with FN and documented bloodstream infection (BSI), although 87% received empirical therapy consistent with IDSA guidelines, 24% were exposed to inadequate initial antimicrobial therapy [2].

Moreover, most infections in patients receiving cancer therapy occur outside of neutropenic episodes, a pattern that is particularly evident among individuals with solid tumors. In a study analyzing 5896 episodes of BSI in patients with solid tumors, only 5.7% were associated with concurrent neutropenia, and the prevalence of multidrug-resistant Gram-negative bacteria (MDR-GNB) more than doubled over a 20-year period [3].

Additionally, an increasing incidence of infections caused by multidrug-resistant organisms (MDROs) has been reported in this population, particularly involving MDR-GNB [4]. A large cohort of outpatients in the USA found that cancer patients had more than twice the risk of harboring an MDRO than general population, particularly carbapenem-resistant or ESBL-producing Gram-negative bacilli [5].

Mortality associated with inappropriate antibiotic therapy in chemotherapy patients is high [6]. Among patients with BSIs, the absence of adequate empirical treatment nearly doubles the risk of death, even after adjusting for potential confounding factors [4]. In a multicenter prospective study evaluating all BSI episodes in high-risk FN patients, mortality was 34% among those who received inappropriate empirical therapy, compared to 24% (*p* = 0.004) in patients who received appropriate antibiotics [2].

Risk factors commonly associated with infections caused by MDROs include prior exposure to antimicrobials, recent hospitalization, healthcare-related interventions, hematologic malignancies, advanced age, infection site, use of invasive devices, and cancer recurrence [7–11]. Nevertheless, in the initial evaluation of an oncologic patient presenting with infection, it is essential to consider a broad differential diagnosis encompassing multidrug-resistant Gram-positive and Gram-negative bacteria, as well as fungal pathogens such as *Candida* spp. Notably, the interactions between different infectious agents remain poorly explored, and few studies have systematically addressed these interactions.

Machine learning has been investigated as a tool to optimize the management of infections in cancer patients, given the need to account for multiple complex factors in this population [12–14]. While predictive models for bloodstream infections (BSIs) have demonstrated good discriminative performance, analyses of risk factors across multiple MDRO types remain less frequent, especially when interactions among these pathogens are explicitly considered.

Therefore, strategies to optimize empirical antimicrobial therapy in cancer patients are essential. This study aims to identify features associated with BSI caused by different groups of MDRO infections using machine learning models applied to a large cohort of patients undergoing chemotherapy.

## Material and methods

### Data source and study population

**Fig. 1:**
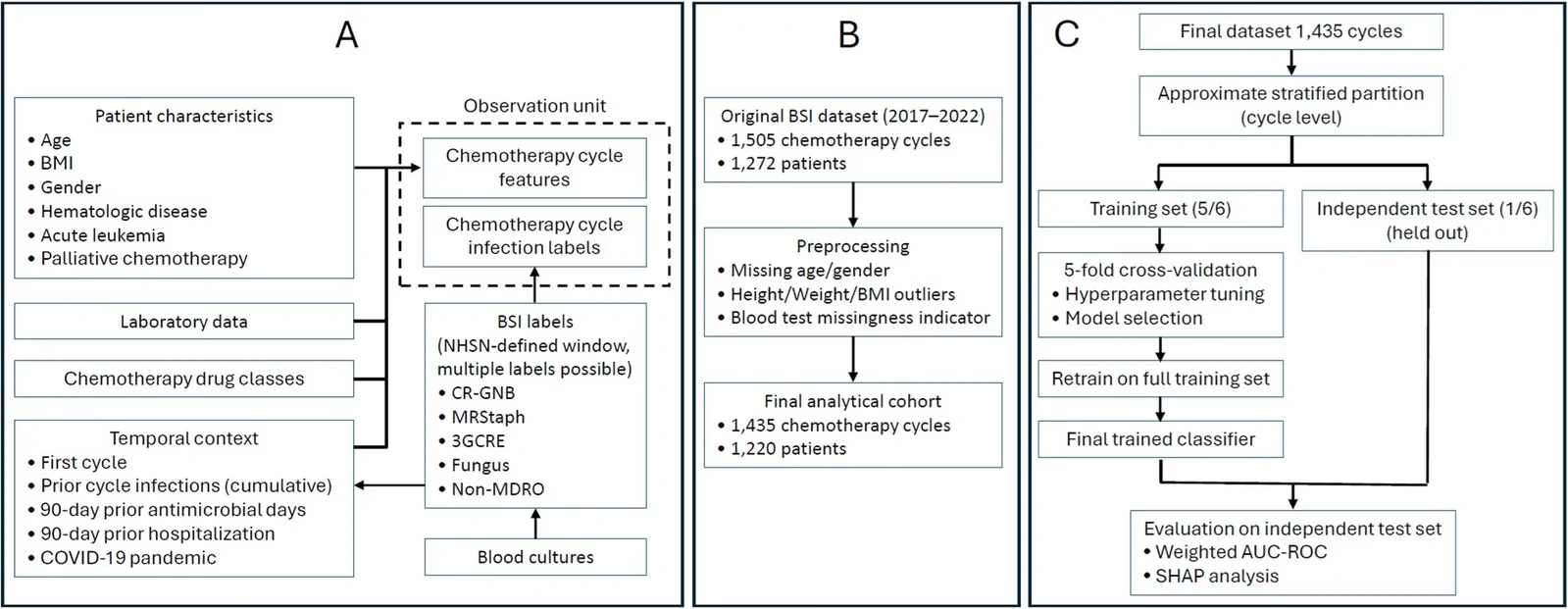
Schematic overview of the analytical workflow. **A** Observation-unit derivation; **B** data preprocessing; and **C** model development and evaluation

This study used a subset of the dataset described in [14], which includes clinical, microbiological, and laboratory data from chemotherapy cycles administered between 2017 and 2022 at the Cancer Institute of the State of Sao Paulo (ICESP), Brazil. Each observation corresponds to a single chemotherapy cycle, and patients may undergo multiple cycles. The original dataset was constructed from electronic medical records and laboratory reports and included variables related to chemotherapy regimens, patient demographics, blood cultures, and laboratory values. The data collected consists of the following information: chemotherapy data (types of cancer, chemotherapy regimens and drugs used, their respective dosages, dates of intravenous administration, treatment schedules followed, as well as weight, height, and body mass index (BMI) at the time of chemotherapy administration), blood cultures (microorganisms and their respective dates), demographic data (date of birth, age, and gender), and blood test results (count of total leukocytes, lymphocytes, neutrophils, monocytes, hematocrit and platelets, and serum creatinine values). Detailed descriptions of the data collection procedures and variable definitions are provided in the Supplementary Material (Appendix A and Table S1). Figure 1A illustrates the derivation of an observation unit, defined as a single analytical instance characterized by a set of explanatory variables and a corresponding multi-label outcome vector, as described below.

For the present analysis, only cycles associated with confirmed BSIs, as defined by NHSN criteria, were included [15]. Each infected cycle was classified according to the microorganism(s) susceptibility profiles. These groups were as follows: (i) carbapenem-resistant Gram-negative bacilli (CR-GNB), (ii) methicillin-resistant staphylococci (MRStaph), (iii) third-generation cephalosporin-resistant Enterobacterales (3GCRE), (iv) fungal infections, and (v) non-MDRO/non-fungal infections. Cycles with polymicrobial infections were classified under all applicable types. These infections groups are not mutually exclusive; there were four infections caused by vancomycin-resistant *Enterococcus* spp in the sample, all of which were polymicrobial. Due to the small number and to avoid overfitting, these infections were classified according to the susceptibility profile of the other agents.

In addition to the variables retained from the original study, new explanatory variables were introduced to capture aspects of patients’ clinical history, recent treatment exposures, and the broader healthcare context. Clinical history was assessed through variables representing the cumulative number of prior infections with each of the five pathogen categories across earlier chemotherapy cycles. Antimicrobial exposure was quantified as the cumulative number of days that each patient received specific antimicrobial agents within the 90-day period preceding the start of each chemotherapy cycle. The antimicrobial classes considered included aminoglycosides, carbapenems, cephalosporins, daptomycin, glycopeptides, linezolid, penicillin/beta-lactamase inhibitors, polymyxins, sulfonamides, tigecycline, amphotericin, anidulafungin, and azoles. Total hospitalization days during the same 90-day window were also recorded. Lastly, to account for potential contextual effects, a binary variable was added to indicate whether the chemotherapy cycle occurred during the COVID-19 pandemic, defined as cycles initiated on or after the last week of February 2020, when institutional protocols were modified in response to the outbreak [16].

Each observation was represented as a static feature vector corresponding to a single chemotherapy cycle, incorporating temporal context directly through variables (a binary indicator for the first cycle, cumulative counts of prior infections, cumulative days of antimicrobial exposure and hospitalization in the preceding 90 days, and a contextual indicator for the COVID-19 pandemic) without modeling full treatment sequences.

### Data preprocessing

The initial data collected for this study included 1505 chemotherapy cycles from 1272 patients, all of which were associated with confirmed BSIs. Data preprocessing included the exclusion of records with missing age or gender, outlier treatment using the interquartile range (IQR) method (Supplementary Material, Appendix B), and the creation of a binary indicator variable (“missing some blood exam”) to flag missing blood test results.

No other variables contained missing values that required exclusion. The IQR method was applied to age, height, weight, and BMI; however, only height, weight, and BMI led to exclusions due to extreme values. After applying all preprocessing steps, the final analytical dataset comprised 1435 cycles from 1220 unique patients. Figure 1B illustrates the derivation of the analysis set through data preprocessing.

### Feature selection method

In this study, we modeled multiple, non-mutually exclusive infection types using a multi-output classification strategy, where predictions for each infection group type could incorporate information from others. Because this approach relies on a shared input feature set across all outcomes, applying outcome-specific feature selection would lead to incompatible models and compromise the structure of interdependent predictions.

To manage model complexity and avoid overfitting, we used regularization and hyperparameter tuning rather than explicit feature selection. For each classification algorithm implemented using Scikit-learn, the following regularization parameters were tuned: maximum tree depth for random forest and gradient boosting algorithms, inverse regularization strength (C) for logistic regression, and the L2 penalty term (alpha) for the multi-layer perceptron (MLP) algorithm [17].

### Data imbalance treatment and scaling

No resampling or reweighting was applied to address class imbalance. All models were trained on the original, imbalanced dataset, allowing predictive performance and feature importance to reflect the true epidemiological distribution of the outcomes.

Feature scaling was applied selectively to numeric variables with large or variable ranges, using the StandardScaler function from Scikit-learn. Specifically, age, BMI, days of hospitalization, and most antimicrobial exposure durations were standardized to have zero mean and unit variance.

No scaling was applied to the cumulative infection history variables, as their values were constrained to a narrow range close to 0–1 and did not warrant further transformation, as well as to the exposure durations for aminoglycosides, daptomycin, and sulfonamides, whose values were already constrained to a 0–1 range.

### Classification

In this study, we employed a multi-label classification framework to predict the infection groups. Each observation could be associated with one or more MDRO groups of infection, allowing for polymicrobial cases. To address this, we followed the approach of Jesse Read et al. and decomposed the multi-label task into a sequence of binary classification problems [18]. Predictions for each label were made sequentially, with the output of preceding labels incorporated as features in subsequent models. This approach enables the model to capture potential dependencies and correlations among outcomes, which is particularly relevant given the possibility of polymicrobial infections. The order of labels in the chain was randomized, and the base classifiers used within these chains were logistic regression, random forests, MLPs, and gradient boosting classifiers. Classifier chains were trained using random label order; performance was averaged over multiple chains to stabilize results.

The latter three models were selected based on their strong predictive performance in our previous study, while logistic regression was included as a reference model for baseline comparison [14]. All models were implemented using the Scikit-learn package for Python.

Hyperparameter tuning focused on model regularization, as described in the *Feature Selection Method* subsection, and chain length. For the MLP algorithm, the network configuration (number of hidden layers and neurons per layer) was also optimized. The tuning process combined grid search with empirically guided refinement (Supplementary Material, Table S2).

### Training, validation, and testing

The dataset (*N* = 1435 cycles) was randomly partitioned a priori into six equally sized subsets at the chemotherapy cycle level (five with 239 observations and one with 240). Five subsets (those with 239 observations each) were combined to form the training set, and the remaining subset (the one with 240 observations) was designated as an independent test set.

Approximate stratification was applied based on infection status. Because cases could involve multiple infection groups, exact stratification across all possible combinations was not feasible. Instead, cases with a single infection were stratified according to the specific infection group present, while all polymicrobial cases were grouped into a single category. This approach approximately preserved the marginal frequency of each infection group and the overall proportion of polymicrobial cases across all data partitions.

Within the training set, fivefold cross-validation was performed for model training, hyperparameter tuning, and internal validation: in each iteration, models were trained on four folds and validated on the remaining fold. The independent test set was fully excluded from all aspects of model tuning and model selection and was used only once to evaluate the final model’s performance and to derive feature contribution metrics. Figure 1C illustrates the model development and evaluation process.

### Model Evaluation and Selection

To evaluate model performance and select the best classifier, we used the weighted area under the receiver operating characteristic curve (AUC-ROC) across the five infection groups. This metric was computed using the *roc_auc_score* function from the Scikit-learn library, with average = “weighted” to account for differences in outcome prevalence.

The AUC-ROC was chosen for its threshold-independent nature, making it particularly well-suited to the study’s main objective of comparing feature importance across models rather than optimizing for specific classification thresholds. Accordingly, threshold-dependent metrics such as F1-score, recall, precision, and specificity were not necessary, as the study did not aim to define or evaluate performance at a particular decision threshold. In addition, AUC-ROC provides a measure of a model’s discriminative ability across the unequal distribution of infection groups in our cohort.

For the final evaluation on the held-out test set, the weighted AUC-ROC was used to assess overall model performance, while AUC-ROC and average precision (AP), which summarizes performance across the precision–recall curve, were additionally reported for each individual infection group as complementary metrics.

## Results

### Descriptive statistics

During the study period (and after the data preprocessing step), we identified 1435 episodes of BSI, corresponding to 1.33% of all chemotherapy cycles among 1220 unique patients. The majority of BSIs occurred during the first cycle of chemotherapy (60.9%). The most frequently used class of chemotherapy drugs was alkylating agents (60.7%). Neutropenia (≤ 500 cells/µL) was present in 30.9% of BSI episodes and lymphopenia (≤ 500 cells/µL) occurred in 52.6% of cases. Full descriptive statistics, stratified by infection group, are presented in Table 1 and Supplementary Table S3.

**Table 1:** Descriptive characteristics of observations from patients undergoing chemotherapy for malignant diseases. Each observation represents the follow-up of one chemotherapy cycle (thus, each patient may be represented more than once). Characteristics are presented overall and stratified by infection type. Because polymicrobial infections may occur, individual observations may be represented in more than one infection category

Characteristics		Overall (*N* = 1435)	CR-GNB (*N* = 109)	MRStaph (*N* = 103)	3GCRE (*N* = 49)	Fungal (*N* = 101)	Non-MDRO/non-fungal (*N* = 1193)
Demographic							
Age (years)	Median	57.43	50.31	55.23	59.50	56.15	57.78
	IQR	43.90–66.34	35.27–62.65	40.73–65.60	42.89–66.27	39.56–65.96	45.02–66.48
BMI (kg/m^2^)	Median	23.78	23.64	25.32	23.66	23.74	23.78
	IQR	20.84–27.18	20.69–26.21	22.45–28.20	19.72–27.07	19.35–26.98	20.92–27.18
Male	*N*	756	64	61	22	42	622
	%	52.7	58.7	59.2	44.9	41.6	52.1
Blood test results							
Creatinine (mg/dL)							
< 1.2	*N*	483	30	41	19	31	392
	%	33.7	27.5	39.8	38.8	30.7	32.9
Hematocrit (%)							
≤ 20	*N*	348	52	24	19	26	269
	%	24.3	47.7	23.3	38.8	25.7	22.6
Lymphocyte (count/µL)							
≤ 500	*N*	755	72	49	34	53	619
	%	52.6	66.1	47.6	69.4	52.5	51.9
Monocyte (count/µL)							
≤ 100	*N*	696	70	50	30	55	559
	%	48.5	64.2	48.5	61.2	54.5	46.9
Neutrophil (count/µL)							
< 200	*N*	407	46	29	19	32	324
	%	28.4	42.2	28.2	38.8	31.7	27.2
Platelet (count 10^9^/L)							
≤ 20	*N*	296	39	23	17	27	230
	%	20.6	35.8	22.3	34.7	26.7	19.3
Chemotherapy regimens							
First cycle	*N*	874	78	67	31	74	714
	%	60.9	71.6	65.0	63.3	73.3	59.9
Palliative treatment	*N*	676	27	38	21	42	589
	%	47.1	24.8	36.9	42.9	41.6	49.4
Chemotherapy classes							
Alkylating agent	*N*	871	54	53	21	69	735
	%	60.7	49.5	51.5	42.9	68.3	61.6
Antimetabolite	*N*	730	55	49	34	51	608
	%	50.9	50.5	47.6	69.4	50.5	51.0
Antitumor antibiotic	*N*	400	47	37	18	27	317
	%	27.9	43.1	35.9	36.7	26.7	26.6
Microtubule inhibitor	*N*	474	42	36	11	40	386
	%	33.0	38.5	35.0	22.5	39.6	32.4
Type of cancer							
Hematologic	*N*	553	69	55	27	38	428
	%	38.5	63.3	53.4	55.1	37.6	35.9
Solid tumor	*N*	882	40	48	22	63	765
	%	61.5	36.7	46.6	44.9	62.4	64.1
COVID-19 epidemic	*N*	699	50	47	19	31	598
	%	48.7	45.9	45.6	38.8	30.7	50.1
Previous conditions							
CR-GNB infection	*N*	24	16	0	0	1	12
	%	1.7	14.7	0.0	0.0	1.0	1.0
Methicillin-R staphylococci infection	*N*	30	3	9	2	3	17
	%	2.1	2.75	8.7	4.1	3.0	1.4
Non-MDRO/non-fungal infection	*N*	222	22	5	7	12	197
	%	15.5	20.2	4.9	14.3	11.8	16.5
Hospitalization (days)	Median	4	9	5	4	6	4
	IQR	0–14	3–24	0–15	0–12	0–15	0–13
Carbapenem antibiotic (days)	Median	0	0	0	0	0	0
	Max	81	17	39	23	27	81
Glycopeptide antibiotic (days)	Median	0	0	0	0	0	0
	Max	28	18	14	27	20	28
30-day mortality	*N*	465	58	31	15	41	376
	%	32.4	53.2	30.1	30.6	40.6	31.5

*CR-GNB* carbapenem-resistant Gram-negative bacteria, *MRStaph* methicillin-resistant staphylococci, *3GCRE* third-generation cephalosporin-resistant *Enterobacterales,* *MDRO* multidrug-resistant organism

Of these episodes, 11.6% (166) were polymicrobial, resulting in a total of 1640 microorganisms isolated. The most frequently identified species was *Escherichia coli* (26.3%), followed by *Klebsiella pneumoniae* (15.5%) and *Pseudomonas aeruginosa* (9.0%) (Supplementary Material, Table S4). Regarding resistance, 6.6% (53) of Enterobacterales isolates were resistant to third-generation cephalosporins and 4.7% (38) were resistant to carbapenems. Overall, carbapenem resistance was observed in 10.8% (114) of Gram-negative bacteria, representing 7.6% (109) of all BSI episodes (Table 2). Thirty-day mortality ranged from 30.1% to 40.6% across the infection groups but was substantially higher in the CR-GNB group, reaching 53.2%.

**Table 2:** Distribution of 1640 microorganisms isolated from 1435 bloodstream infections and susceptibility profile of pathogens of interest

Microorganism	Number
Enterobacterales	806
3rd-generation cephalosporin-resistant*	53 (6.6%)
Carbapenem-resistant*	38 (4.7%)
Non-fermentative Gram-negative	217
Carbapenem-resistant*	67 (31.0%)
Other Gram-negative	34
Carbapenem-resistant*	9 (26.5%)
*S. aureus*	115
Methicilin-resistant*	40 (34.8%)
Coagulase-negative staphylococci	69
Methicillin-resistant*	63 (91.3%)
Other Gram-positive	215
Anaerobic	81
Fungal	103

^*^Proportion relative to the total number of microorganisms in this group

Preliminary analysis of the correlation matrix among the five infection groups revealed negative correlations between non-MDRO/non-fungal infections and each of the three MDRO groups, as well as fungal infections (Supplementary Table S5). Because most BSI episodes in the cohort were monomicrobial, these negative correlations are expected, as monomicrobial episodes cannot simultaneously include organisms belonging to different infection groups. To further examine this issue, we repeated the analysis after restricting the dataset to the 114 episodes with more than one susceptibility profile group, in which organisms from different infection groups may coexist (Supplementary Table S6). In this subset, the negative correlations involving non-MDRO/non-fungal infections were substantially attenuated, indicating that much of the overall pattern reflects the predominance of monomicrobial infections.

### Overview of findings

**Fig. 2:**
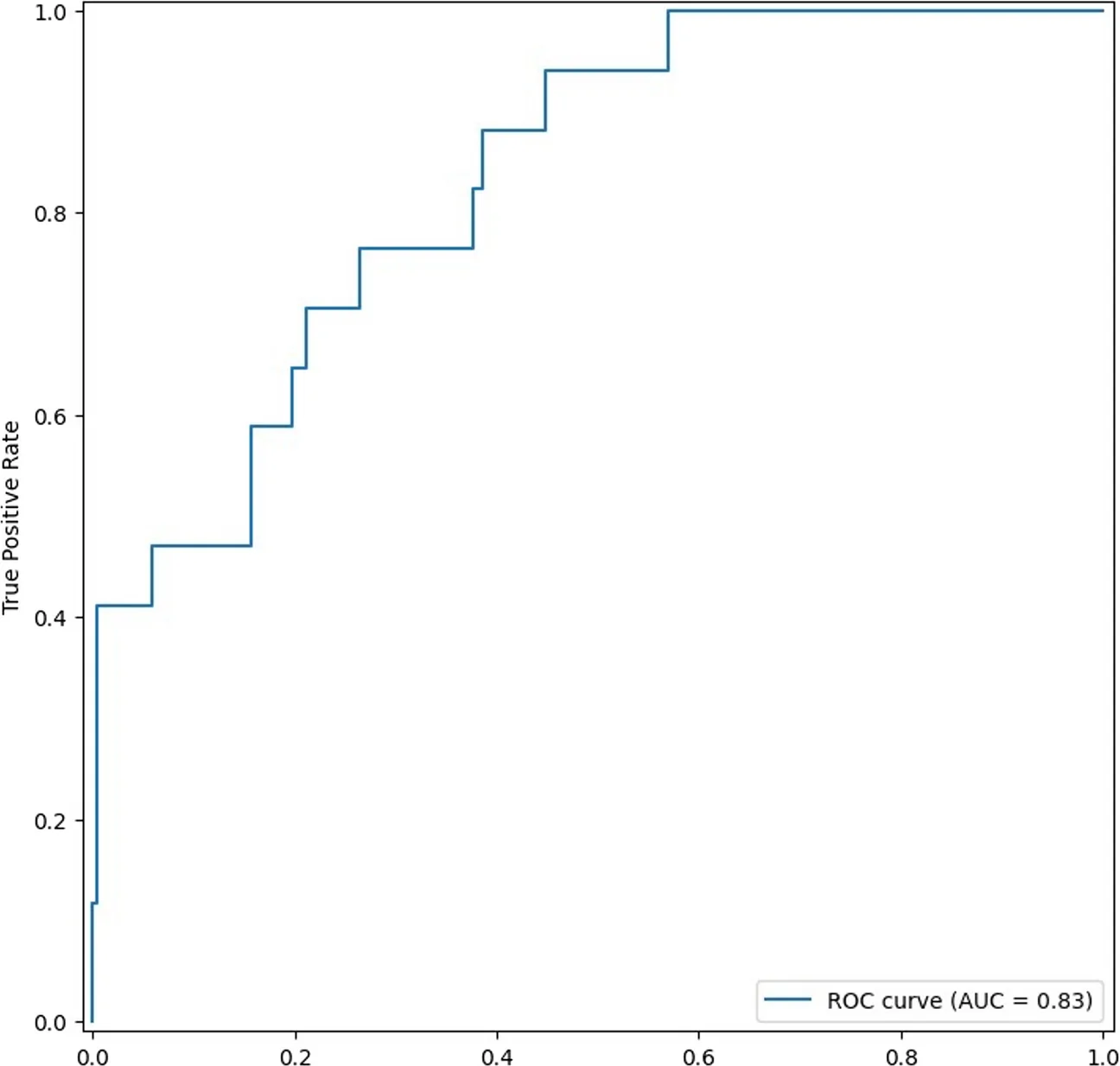
ROC curve for the best model (chain of 20 random forests of 100 trees with maximum depth of 4), evaluated on the test set for carbapenem-R Gram-negative bacilli infection

The best-performing model (highest average AUC-ROC across infection types, weighted by the number of positive cases) was a random forest classifier with 100 trees, a maximum depth of 4, and implemented within a classifier chain of length 20. It achieved a weighted average AUC-ROC of 60.90% with a standard deviation (SD) of 2.52% across infection types in cross-validation. On the held-out test set, the weighted average AUC-ROC was 66.37% (Fig. 2 and Supplementary Material, Figures S1–S4).

The AUC-ROC results for each infection type (Table 3) show that, except for fungal infections, the test set AUC-ROC values were higher than those obtained in cross-validation. This pattern was most pronounced for CR-GNB, where the AUC-ROC increased from 66.05% in cross-validation to 83.25% in the test set (Fig. 2). The corresponding AP values on the held-out test set range from 6.40% for 3GCRE to 90.84% for non-MDRO/non-fungal, with differences across infection types reflecting, in part, differences in prevalence (Table 3 and Supplementary Material, Figures S5–S9).

**Table 3:** Cross-validation and test set results across infection types for the random forest classifier with 100 trees, maximum depth of 4, and implemented within a classifier chain of length 20

Infection	Cross-validation		Test set		
	Mean AUC-ROC	Standard deviation	*N* (%)	AUC-ROC (95% CI)	AP (95% CI)
CR-GNB	66.05%	7.45%	17 (7.1)	83.25% (73.91%–91.64%)	47.38% (24.09%–70.41%)
MRStaph	57.16%	7.33%	18 (7.5)	63.06% (46.84%–77.01%)	16.50% (7.60%–36.00%)
3GCRE	56.12%	21.59%	8 (3.3)	62.61% (37.83%–83.48%)	6.40% (2.23%–18.96%)
Fungal	61.05%	2.65%	18 (7.5)	51.75% (36.98%–66.21%)	11.64% (5.39%–27.56%)
Non-MDRO/non-fungal	60.83%	2.35%	199 (82.9)	66.70% (57.42%–74.75%)	90.84% (86.71%–94.29%)

*CR-GNB* carbapenem-resistant Gram-negative bacteria, *MRStaph* methicillin-resistant staphylococci, *3GCRE* third-generation cephalosporin-resistant Enterobacterales, *MDRO* multidrug-resistant organism, *AUC-ROC* area under the receiver operating characteristic curve, *AP* average precision based on the area under the precision–recall curve, *CI* confidence interval. The 95% CIs for AUC-ROC and AP were estimated using nonparametric bootstrap resampling of the independent test set with 2000 bootstrap replicates

The most influential features were days of hospitalization, palliative chemotherapy, hematologic disease, previous CR-GNB infection, and monocytopenia.

### Performance of the models

Among the best-performing parameterizations evaluated for each model type (Supplementary Table S7), the random forest classifier achieved the highest weighted average AUC-ROC (60.90%, SD = 2.52%) across infection types when implemented within a classifier chain of length 20. The neural network (59.96%, SD = 2.89%) and logistic regression (59.96%, SD = 2.81%) models showed similar performance, while the gradient boosting model performed slightly worse (56.89%, SD = 1.03%). However, given the magnitude of the standard deviations relative to the differences in mean AUC-ROC, none of the models demonstrated statistically meaningful superiority.

### Feature contributions

**Fig. 3:**
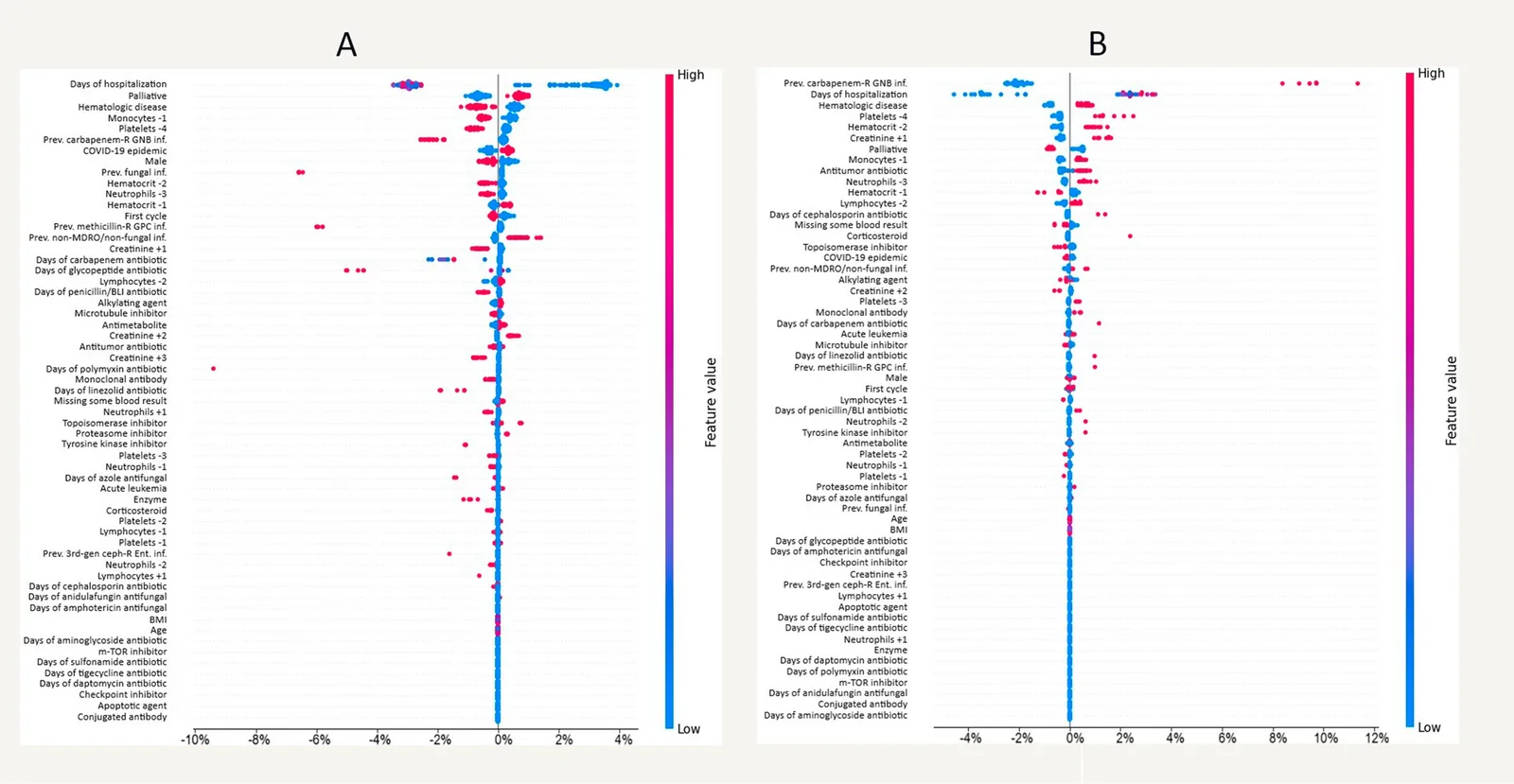
Shapley values for the best model (chain of 20 random forests of 100 trees with maximum depth of 4), evaluated on the test set for **A** non-multidrug-resistant organism/non-fungal infection and **B** carbapenem-R Gram-negative bacilli infection, computed using the SHAP package in Python

We assessed model explanations using SHapley Additive exPlanations (SHAP) values derived from the test set (Fig. 3 and Supplementary Material, Figures S10–S12) [19]. Among the 59 candidate variables, the 20 most influential features were selected based on SHAP values. The variables with the greatest overall contribution were days of hospitalization, palliative chemotherapy, hematologic malignancy, monocytopenia, and thrombocytopenia (Fig. 3).

**Fig. 4:**
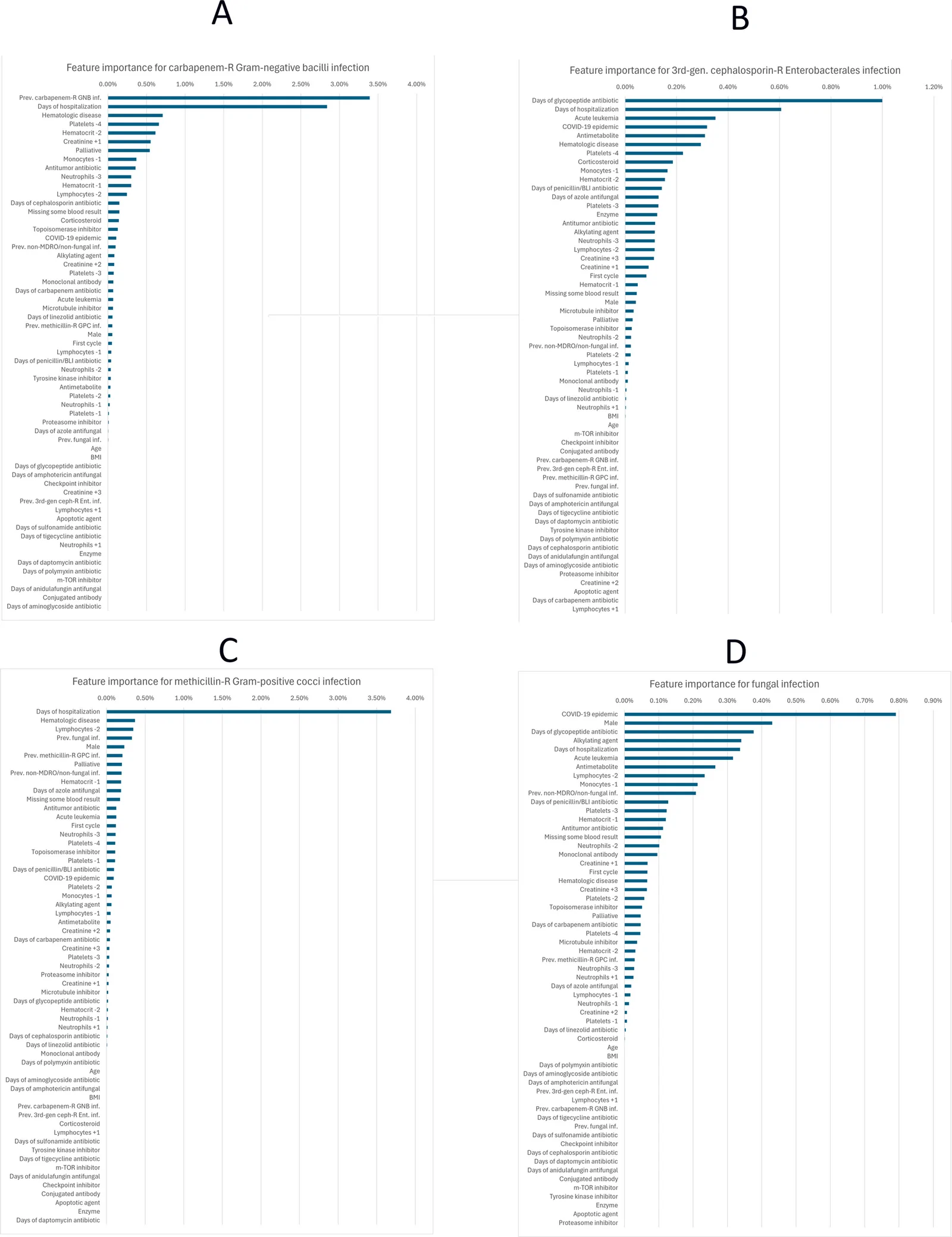
Mean absolute Shapley values for the best model (chain of 20 random forests of 100 trees with maximum depth of 4), evaluated on the test set and computed using the SHAP package in Python. **A** Carbapenem-R Gram-negative bacilli, **B** 3rd-gen. cephalosporin-R Enterobacterales, **C** methicillin-R staphylococci, and **D** fungal

Among the top-ranked features shown in Fig. 4 and Table 4, the most important predictors for CR-GNB infection were previous CR-GNB infection, days of hospitalization, hematologic malignancy, thrombocytopenia, and low hematocrit. For 3GCRE, the highest-ranking features were previous glycopeptide exposure, days of hospitalization, acute leukemia, infection during COVID-19 pandemic (which was negatively associated), and the use of antimetabolites in the chemotherapy regimen. For MRStaph, the most important features were days of hospitalization, hematologic malignancy, lymphopenia, previous fungal infection, and male sex. For fungal infections, the highest-ranking features were the COVID-19 pandemic period, male gender, days of glycopeptide use, alkylating agent in the chemotherapy regimen, and days of hospitalization (see Supplementary Material, Figures S13–S14, for complementary information on overall feature importance and the non-MDRO/non-fungal outcome).

**Table 4:**
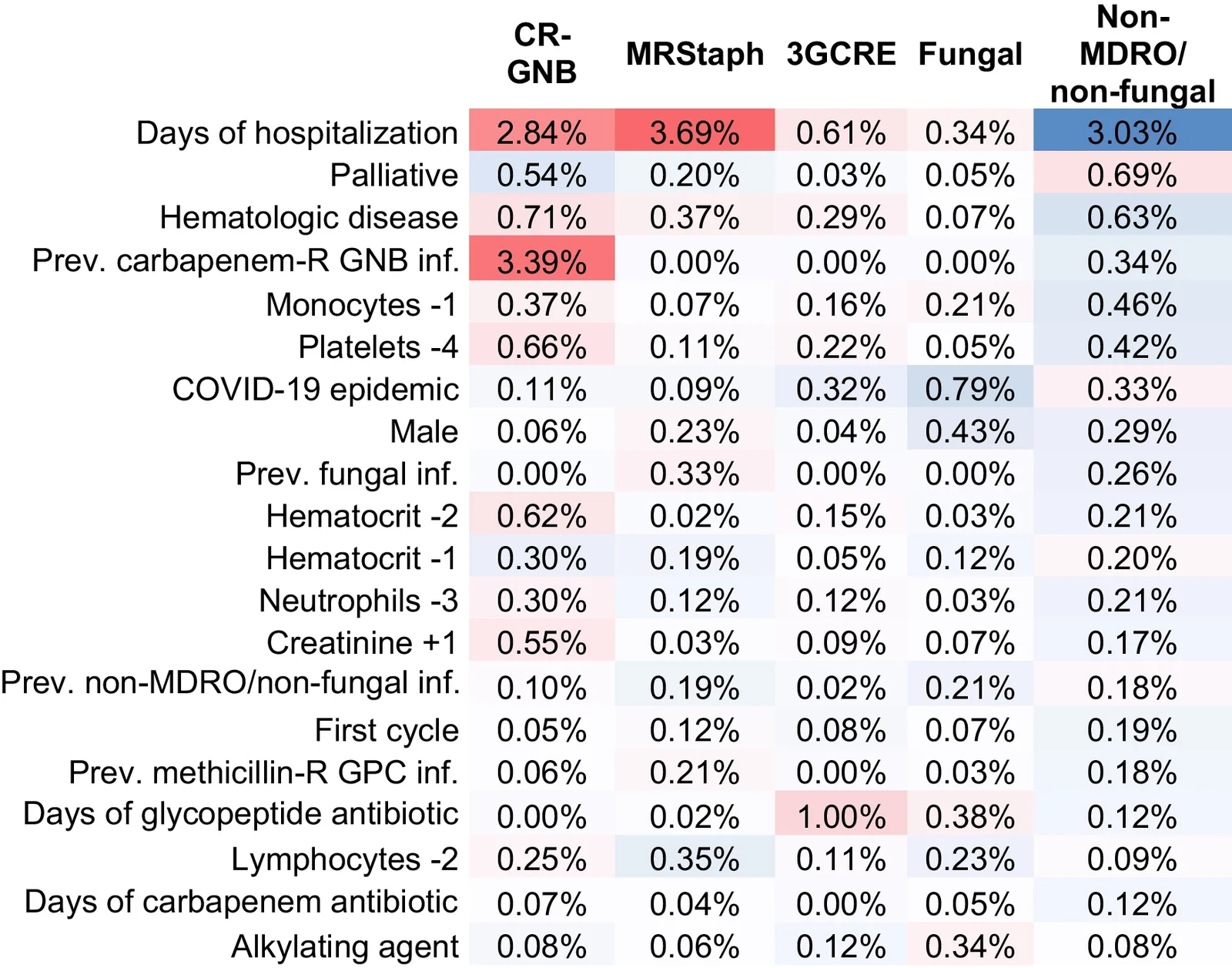
Top 20 features, ranked by their overall SHAP importance (computed as a weighted average across infections, weighting by the number of positive cases). Each cell shows the mean absolute SHAP value for that feature within a specific infection type. SHAP values are derived from the test set. Legend: Red shading indicates variables associated with higher predicted probability, while blue shading indicates variables associated with lower predicted probability, with color intensity reflecting magnitude

To illustrate how specific chemotherapy regimens may be associated with each infection group, Table 5 presents the SHAP-based ranking of chemotherapy class features only. These values were calculated using the best-performing model, controlling for all other variables. The top three ranked classes were alkylating agents, antitumor antibiotics, and antimetabolites.

**Table 5:**
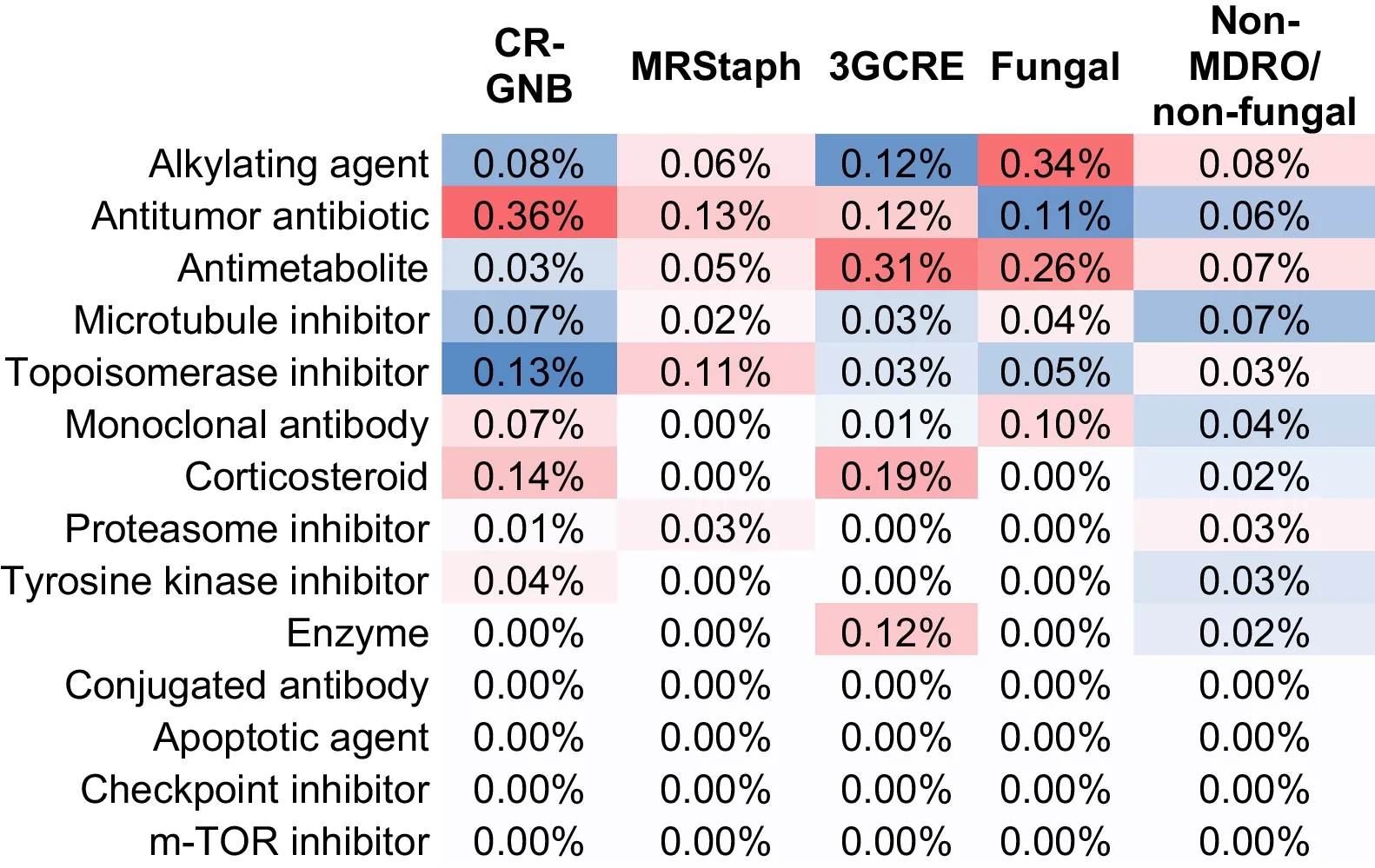
Chemotherapy class features, ranked by their overall SHAP importance (computed as a weighted average across infections, weighting by the number of positive cases). Each cell shows the mean absolute SHAP value for that feature within a specific infection type. SHAP values are derived from the test set. Legend: Red shading indicates variables associated with higher predicted probability, while blue shading indicates variables associated with lower predicted probability, with color intensity reflecting magnitude

## Discussion

This study analyzed a large database of patients who underwent chemotherapy over a 5-year period, aiming to explore whether multi-label machine learning could identify clinical factors associated with different bloodstream infection (BSI) etiologies in cancer patients. The goal was to generate hypotheses that could eventually support clinical decision-making about when to use standard empirical therapy for patients with suspected infections and when broader antimicrobial coverage might be necessary, particularly in settings where multiple infection types may coexist. By modeling the potential interdependence between pathogen groups, the approach aimed to reflect the clinical reality of polymicrobial infections. Additionally, our analysis suggested that specific chemotherapy classes may be differentially associated with particular infection groups, although these associations require external validation.

Several studies have shown that cancer patients with MDRO infections are at increased risk of receiving inappropriate empirical antimicrobial therapy, highlighting the importance of models to guide treatment in patients with suspected BSI [2, 20].

In our cohort, MDRO BSI accounted for 17.8% of episodes, with high mortality rate, especially for CR-GNB. The most influential features were those typically linked to a higher MDRO risk (such as prolonged hospitalization, hematologic malignancies, and prior MDRO isolation); because the analysis was restricted to chemotherapy cycles with confirmed BSIs, these features were associated with a higher predicted probability of MDRO infection and a lower predicted probability of non-MDRO/non-fungal infection in this relative setting (Table 4 and Supplementary Material, Figure S13) [3, 21, 22].

Little is known about the role of different chemotherapy types in the risk of MDRO infection. This is likely because, in immunocompromised patients, MDRO risk is mainly driven by prolonged or recurrent infections, multiple or broad-spectrum antibiotic courses, and healthcare exposure. However, chemotherapy itself affects the microbiome and may influence MDRO dynamics; for example, purine analogues have been described as adjuvants that resensitize MRSA to beta-lactam antibiotics [23]. Therefore, it is plausible that different chemotherapy classes confer distinct MDRO risks, especially when adjusted for established risk factors.

Chemotherapy agents demonstrated an overall lower contribution to the machine learning models for predicting infection types than other features, such as duration of hospitalization, prior MDRO isolation, and the presence of hematologic malignancy. Nonetheless, certain associations between specific chemotherapy classes and microbial groups warrant further consideration, although these associations remain largely speculative. (Table 5).

Antimetabolites were associated with an increased probability of infection by 3GCRE. These cytotoxic agents disrupt nucleotide metabolism and can suppress bone marrow function, having previously been linked to a higher risk of FN compared to other chemotherapy classes [24]. Additionally, their relationship with the microbiome is complex: antimetabolites commonly reduce microbial diversity and promote the overgrowth of opportunistic bacteria such as *E. coli* (Proteobacteria) [25].

Antitumor antibiotic was associated with an increased probability for CR-GNB BSI. Although these agents do not directly target bacteria or exert selective pressure in the same manner as antimicrobial agents, they may contribute to CR-GNB infections through several mechanisms, such as disrupting the gut microbiome, inducing neutropenia, and causing mucosal barrier injury [26]. Chemotherapy may also increase the basal mutation rate in bacteria, enhancing the selection of antibiotic-resistant mutants. One study reported that exposure to daunorubicin and mitoxantrone induced mutations in Enterobacterales, raising the MIC of ceftazidime-avibactam to 64 and 32 mg/L, respectively, compared to 4 and 1 mg/L in untreated controls [27].

Alkylating agents were associated with candidemia. This association likely results from a combination of profound neutropenia induced by certain regimens (e.g., those including cyclophosphamide or busulfan) and mucosal damage caused by agents such as melphalan and cyclophosphamide [28].

Although monocytopenia has not been specifically linked to MDRO infection, some studies have associated it with an increased overall risk of infection [29]. In allogeneic stem cell transplant recipients, lower monocyte counts were inversely associated with infection risk, underscoring the importance of monocyte reconstitution in infection control among immunocompromised hosts [30]. In our cohort, palliative chemotherapy was positively associated with the indication for standard empirical therapy (Table 4), a finding that should be interpreted relative to other cancer patients undergoing chemotherapy. Palliative regimens are generally less intensive, involving fewer invasive procedures, device use, and side effects such as mucositis [31]. This lower treatment intensity may also limit microbiota disruption, potentially reducing the risk of MDRO infections [26].

The COVID-19 pandemic period emerged as the most important feature associated with fungal infections during chemotherapy. Increased rates of candidemia have been reported during the pandemic, likely reflecting multiple changes in healthcare delivery rather than a direct effect of SARS-CoV-2 infection itself. Although our models adjusted for prior broad-spectrum antimicrobial exposure and most patients received chemotherapy in the outpatient setting, the pandemic period may represent a surrogate marker for unmeasured changes in healthcare exposure, clinical practice and indiscriminate antimicrobial use [32].

Previous antimicrobial use has been commonly reported as a risk factor for MDRO infections [33]. In our study, we included several antimicrobial classes as candidate predictors; however, overall, their associations with infection risk were relatively weak. One noteworthy finding was the association between prior glycopeptide use and infection caused by 3GCRE or fungi.

Our main hypothesis is that prior glycopeptide use is a marker of greater healthcare exposure rather than a specific risk factor for 3GCRE or fungal infections. As glycopeptides are generally reserved for suspected or documented Gram-positive infections and are not routinely used for uncomplicated infections in patients undergoing chemotherapy, their use likely identifies patients at increased risk of infection overall rather than of these specific pathogens.

For CR-GNB BSI (Fig. 3B), prior CR-GNB infection was the strongest associated feature. Patients with a previous CRE infection were presumed to remain colonized, and CRE colonization in patients with cancer has been shown to increase the risk of subsequent BSI by nearly fourfold (13–22% incidence among colonized) [34–38]. Despite this, guidelines remain divided on systematic empirical coverage, given concerns over broad-spectrum antibiotic overuse and resistance. Low hematocrit and platelet counts, likely markers of severe myelosuppression, also predicted CR-GNB BSI. These findings suggest that markers of hematologic impairment, potentially reflecting systemic inflammation or frailty, may help refine empirical therapy decisions in high-risk cancer patients [39–41].

The modest AUC-ROC values observed in this study may be attributed, in part, to the limited discriminative power of individual predictors. More importantly, however, the performance likely reflects the restricted size of the dataset used to train the models. Machine learning algorithms, particularly those designed to capture non-linear relationships, typically require large volumes of data of all classes to detect and generalize complex patterns effectively. This presents a common limitation in clinical infection modeling, where the number of infected cases is inherently constrained due to active infection control measures in routine care, which reduce the prevalence of such outcomes. The higher test set performance may therefore reflect sampling variability associated with the relatively small number of cases in some outcome groups, as suggested by the wide 95% confidence intervals around the AUC-ROC estimates (Table 3). Accordingly, discrimination estimates for the rarer infection groups should be interpreted with caution because of the limited number of positive events in the independent test set.

To address the previously mentioned possibility that correlations among infection types may influence model performance, we compared the classifier chain approach with its independent counterpart. The best model (a random forest classifier with 100 trees, a maximum depth of 4, implemented within a classifier chain of length 20) performed only marginally better than its independent version (a random forest with 2000 trees, fitting separate binary classifiers without chaining, thereby assuming no dependency among the labels) in cross-validation (weighted average AUC-ROC of 60.86%, SD = 2.93%). This difference is not statistically meaningful, and the results provide no evidence that exploiting label dependencies through classifier chaining improves predictive performance on this dataset. Nevertheless, this should not preclude further exploration of methods that incorporate label dependencies in multi-label settings in similar studies.

Since a major goal of the study is to assess the relative importance of predictors across infection types of groups, our choice of retaining the full set of explanatory variables avoids prematurely excluding features whose relevance may depend on interactions or model architecture. This also ensures that importance scores reflect the entire clinical context, rather than a filtered subset.

The best-performing model, based on a non-linear algorithm, did not significantly outperform the logistic regression model, which is linear (Supplementary Table S7). Nevertheless, exploring non-linear relationships in this context may be valuable for improving predictive performance and deepening our understanding of features associated with infection.

This study has some limitations. It is a single-center study, and the model needs to be validated in other institutions with different oncologic patient profiles and MDR bacterial profiles. The study period included the COVID-19 pandemic, which altered healthcare delivery across institutions and affected the epidemiology of MDRO infections. To minimize this bias, we included the pandemic period as an adjustment variable. Despite the large initial cohort, the number of BSIs and MDR-related BSIs was relatively small, as bacteremia during chemotherapy is an uncommon event, limiting predictive performance and contributing to high variability in some outcome groups. Because this study focused on the chemotherapy level, we partitioned the data accordingly. However, patient-level partitioning would provide a more conservative assessment of model generalizability when evaluating performance on previously unseen patients. Moreover, fungal infections comprised a small number of cases and had the lowest AUC-ROC; therefore, associations with this group should be interpreted with caution. Finally, the retrospective design limits causal inference, and associations observed here should be considered hypothesis-generating.

In conclusion, our findings underscore the complexity of infection risk in the cancer population and highlight the relevance of host-related features such as hematologic malignancy, prior MDRO infection, and cytopenias, as well as the potential influence of specific chemotherapy classes. Despite the challenges in building models for uncommon events such as bloodstream infections, the multi-label modelling approach proved feasible and generated exploratory insights that may inform future studies and risk stratification efforts in these immunocompromised populations.

## Supplementary information

Below is the link to the electronic supplementary material.

ESM 1 (DOCX 1.63 MB)

## Data Availability

The datasets generated and/or analyzed during the current study are not publicly available due to due to confidentiality concerns but are available from the corresponding author on reasonable request.
