# Supplementary material for: Application of machine learning to identify risk factors for bloodstream infections caused by different antimicrobial resistance phenotypes in chemotherapy patients
Source: Support Care Cancer. 2026 Oct 20;34(11):1127. doi: 10.1007/s00520-026-11379-2 (PMC13683477; doi:10.1007/s00520-026-11379-2)
Supplement: Supplementary file 1 [file 520_2026_11379_MOESM1_ESM.docx]

# Supplementary material

# Appendix A - Data collection procedures and variable definitions

The data were collected from chemotherapy treatments between 2017 and 2022 at the Cancer Institute of the State of Sao Paulo (ICESP), Brazil. Each observation period corresponds to the follow-up of one patient's cycle, meaning the same patient could be included more than once.

The initial database was generated from patients’ electronic medical records and laboratory reports. The electronic records included specific fields for chemotherapy treatment, as well as daily clinical evaluations and vital signs during chemotherapy administration.

The data collected consists of the following information: chemotherapy data (types of cancer, chemotherapy regimens and drugs used, their respective dosages, dates of intravenous administration, treatment schedules followed, as well as weight, height, and Body Mass Index (BMI) at the time of chemotherapy administration), blood cultures (microorganisms and their respective dates), demographic data (date of birth, age, and gender) and blood test results (count of total leukocytes, lymphocytes, neutrophils, monocytes, hematocrit and platelets, and serum creatinine values). We converted the numerical results into ranges, based on cut-offs previously used in other scores (CISNE, APACHE III, SOFA).

Binary variables were created for the following information: first cycle, palliative chemotherapy, and drugs, including those that should be administered orally, converted into classes. The chemotherapy classes used were alkylating agent, antitumor antibiotic, conjugated antibody, monoclonal antibody, antimetabolite, corticosteroid, checkpoint inhibitor, microtubule inhibitor, proteasome inhibitor, topoisomerase inhibitor, enzyme, tyrosine kinase inhibitor, mTOR inhibitor, apoptotic agent, and SUMOylation inhibitor.

For the outcome BSI in each cycle, the association window between microbial cultures and the chemotherapy treatment cycle was 21 days (or 30 days for acute leukemia) after any date of intravenous medication administration. If two or more successive cycles matched the same positive culture, the culture was associated only with the earliest cycle. BSI was defined according to National Healthcare Safety Network (NHSN-CDC) criteria, therefore, commensal pathogens were considered only in the presence of fever and two distinct positive blood cultures.

The association window between blood tests and the chemotherapy cycle was 21 days after the first administration of intravenous medication in the cycle. When multiple blood test results were available during the observation period, the lowest values for lymphocytes, neutrophils, monocytes, hematocrit, and platelets, and the highest value for creatinine were selected. If the patient had a positive blood culture during this period, the blood test association window ended on the date of the first positive blood culture.

**Appendix B - Outlier treatment**

Outlier treatment was performed using the Interquartile Range (IQR) method for continuous variables. The lower quartile (Q1) corresponds to the 25th percentile, and the upper quartile (Q3) corresponds to the 75th percentile. A multiplicative factor of 1.5 was applied to exclude observations with continuous variables falling outside the range of Q1 - 1.5 × (Q3 - Q1) to Q3 + 1.5 × (Q3 - Q1).

| Supplementary Table S1 - Reference table to convert blood test results into ranges. Numerical labels for ranges specify how much below or above the level is with respect to the normal range used in the developing of a machine learning model to predict the occurrence of a bloodstream infection during chemotherapy | | | | | | | | |
| --- | --- | --- | --- | --- | --- | --- | --- | --- |
|  | **Range** | | | | | | | |
|  | **- 4** | **- 3** | **- 2** | **- 1** | **Normal** | **+ 1** | **+ 2** | **+ 3** |
| **Lymphocytes**  **(Count/Nanolitres)** |  |  | ≥ 0  ≤ 0.5 | > 0.5  ≤ 1 | > 1  ≤ 3 | > 3 |  |  |
| **Neutrophils**  **(Count/nanolitre)** |  | ≥ 0  ≤ 0.2 | > 0.2  ≤ 0.5 | > 0.5  ≤ 1 | > 1  ≤ 7 | > 7 |  |  |
| **Monocytes**  **(Count/nanolitres)** |  |  |  | ≥ 0  ≤ 0.1 | > 0.1 |  |  |  |
| **Hematocrit**  **(%)** |  |  | ≥ 0  ≤ 20 | > 20  < 30 | ≥ 30  < 46 | ≥ 46  < 50 | ≥ 50  < 60 | ≥ 60 |
| **Platelets**  **(Count/nanolitre)** | ≥ 0  ≤ 20 | > 20  < 50 | ≥ 50  < 100 | ≥ 100  < 150 | ≥ 150 |  |  |  |
| **Creatinine**  **(mg/dL)** |  |  |  |  | ≥ 0  < 1.2 | ≥ 1.2  < 2 | ≥ 2  < 3.5 | ≥ 3.5 |

| Supplementary Table S2 – Tested hyperparameters and configurations in the Scikit-learn package implementation. | |
| --- | --- |
| **Model** | **Hyperparameters and configurations** |
| Classifier chain | Base classifiers = logistic regression, multi-layer perceptron, gradient boosting, and random forest  Chain length: 10, 20, 30, 40, 50, 60, 70, 80, 90, 100, 100, 110, and  120 |
| Logistic regression | LogisticRegression(penalty='l2', C=regul_param, max_iter=100, tol=0.000001, solver='newton-cg')  with regul_param = 0.01, 0.1, 1, and 10 |
| Multi-layer perceptron | MLPClassifier(max_iter=10000, solver='lbfgs', alpha=regul_param, hidden_layer_sizes=h, random_state=1, activation='logistic')  with regul_param = 0.0001, 0.001, 0.01 and 0.1, while h = (2,), (3,),  (5,), (10,), (5,5,), (10,10,), (20,20,), (50,50,), and (100,100,100,) |
| Gradient boosting | GradientBoostingClassifier(n_estimators=100, learning_rate=1.0, max_depth=regul_param, random_state=0)  with regul_param = 1, 2, 3, 4, and 5 |
| Random forest | RandomForestClassifier(random_state=0, max_depth=regul_param, n_estimators=100)  with regul_param = 1, 2, 3, 4, 5, 6, 7, 8, 9, 10, 15, and None |

Supplementary Table S3. Descriptive characteristics of observations from patients undergoing chemotherapy for malignant diseases. Each observation represents the follow-up of one chemotherapy cycle (thus, each patient may be represented more than once). Characteristics are presented overall and stratified by infection type. Because polymicrobial infections may occur, individual observations may be represented in more than one infection category.

| **Characteristics** | | **Overall**  **(N=** **1435)** | **CR-GNB**  **(N=109)** | **MRStaph**  **(N=103)** | **3GCRE**  **(N=49)** | **Fungus**  **(N=101)** | **Non-MDRO/ non-fungal**  **(N=1193)** |
| --- | --- | --- | --- | --- | --- | --- | --- |
| Demographic | |  |  |  |  |  |  |
| Age (years) | Median | 57.43 | 50.31 | 55.23 | 59.50 | 56.15 | 57.78 |
|  | IQR | 43.90- 66.34 | 35.27-62.65 | 40.73-65.60 | 42.89-66.27 | 39.56-65.96 | 45.02-66.48 |
| BMI (Kg/m²) | Median | 23.78 | 23.64 | 25.32 | 23.66 | 23.74 | 23.78 |
|  | IQR | 20.84- 27.18 | 20.69-26.21 | 22.45-28.20 | 19.72-27.07 | 19.35-26.98 | 20.92-27.18 |
| Height (cm) | Median | 164 | 166 | 166 | 163 | 163 | 164 |
|  | IQR | 156-170 | 156-172 | 157-170 | 156-168 | 155-169 | 156-170 |
| Male | N | 756 | 64 | 61 | 22 | 42 | 622 |
|  | % | 52.7 | 58.7 | 59.2 | 44.9 | 41.6 | 52.1 |
| Weight (Kg) | Median | 64.25 | 62.30 | 70.00 | 64.50 | 62.20 | 64.00 |
|  | IQR | 54.68- 74.45 | 54.90-72.80 | 58.00-77.25 | 54.00-71.8 | 52.10-74.40 | 54.80-74.40 |
| Blood test results | |  |  |  |  |  |  |
| Missing  result | N | 471 | 32 | 38 | 11 | 32 | 398 |
|  | % | 32.8 | 29.4 | 36.9 | 22.5 | 31.7 | 33.4 |
| Creatinine (mg/dL) | |  |  |  |  |  |  |
| < 1.2 | N | 483 | 30 | 41 | 19 | 31 | 392 |
|  | % | 33.7 | 27.5 | 39.8 | 38.8 | 30.7 | 32.9 |
| 1. 2 - 2 | N | 212 | 26 | 9 | 7 | 17 | 174 |
|  | % | 14.8 | 23.9 | 8.7 | 14.3 | 16.8 | 14.6 |
| 2 - 3.5 | N | 177 | 11 | 8 | 7 | 10 | 158 |
|  | % | 12.3 | 10.1 | 7.8 | 14.3 | 9.9 | 13.2 |
| ≥ 3.5 | N | 96 | 10 | 7 | 5 | 11 | 75 |
|  | % | 6.7 | 9.2 | 6.8 | 10.2 | 10.9 | 6.3 |
| Hematocrit | |  |  |  |  |  |  |
| ≤ 20 | N | 348 | 52 | 24 | 19 | 26 | 269 |
|  | % | 24.3 | 47.7 | 23.3 | 38.8 | 25.7 | 22.6 |
| 21 - 30 | N | 475 | 19 | 26 | 16 | 39 | 407 |
|  | % | 33.1 | 17.4 | 25.2 | 32.7 | 38.6 | 34.1 |
| 30 - 46 | N | 153 | 6 | 16 | 4 | 4 | 130 |
|  | % | 10.7 | 5.5 | 15.5 | 8.2 | 4.0 | 10.9 |
| 46 - 50 | N | 0 | 0 | 0 | 0 | 0 | 0 |
|  | % | 0.0 | 0.0 | 0.0 | 0.0 | 0.0 | 0.0 |
| 50 - 60 | N | 0 | 0 | 0 | 0 | 0 | 0 |
|  | % | 0.0 | 0.0 | 0.0 | 0.0 | 0.0 | 0.0 |
| > 60 | N | 0 | 0 | 0 | 0 | 0 | 0 |
|  | % | 0.0 | 0.0 | 0.0 | 0.0 | 0.0 | 0.0 |
| Lymphocyte (count/µL) | |  |  |  |  |  |  |
| ≤ 500 | N | 755 | 72 | 49 | 34 | 53 | 619 |
|  | % | 52.6 | 66.1 | 47.6 | 69.4 | 52.5 | 51.9 |
| 501 - 1000 | N | 132 | 3 | 9 | 4 | 9 | 110 |
|  | % | 9.2 | 2.8 | 8.7 | 8.2 | 8.9 | 9.2 |
| 1000 - 3000 | N | 83 | 2 | 8 | 1 | 6 | 72 |
|  | % | 5.8 | 1.8 | 7.8 | 2.0 | 5.9 | 6.0 |
| > 3000 | N | 6 | 0 | 0 | 0 | 1 | 5 |
|  | % | 0.4 | 0.00 | 0.00 | 0.00 | 0.99 | 0.42 |
| Monocyte (count/µL) | |  |  |  |  |  |  |
| ≤ 100 | N | 696 | 70 | 50 | 30 | 55 | 559 |
|  | % | 48.5 | 64.2 | 48.5 | 61.2 | 54.5 | 46.9 |
| > 100 | N | 280 | 7 | 16 | 9 | 14 | 247 |
|  | % | 19.5 | 6.4 | 15.5 | 18.4 | 13.9 | 20.7 |
| Neutrophil (count/µL) | |  |  |  |  |  |  |
| < 200 | N | 407 | 46 | 29 | 19 | 32 | 324 |
|  | % | 28.4 | 42.2 | 28.2 | 38.8 | 31.7 | 27.2 |
| 200 - 500 | N | 37 | 5 | 3 | 2 | 3 | 32 |
|  | % | 2.6 | 4.6 | 2.9 | 4.1 | 3.0 | 2.7 |
| 500 - 1000 | N | 125 | 9 | 11 | 1 | 8 | 102 |
|  | % | 8.7 | 8.3 | 10.7 | 2.0 | 7.9 | 8.6 |
| 1000 - 7000 | N | 308 | 13 | 17 | 14 | 16 | 269 |
|  | % | 21.5 | 11.9 | 16.5 | 28.6 | 15.8 | 22.6 |
| > 7000 | N | 99 | 4 | 6 | 3 | 10 | 79 |
|  | % | 6.9 | 3.7 | 5.8 | 6.1 | 9.9 | 6.6 |
| Platelet (count 10^9^/L) | |  |  |  |  |  |  |
| ≤ 20 | N | 296 | 39 | 23 | 17 | 27 | 230 |
|  | % | 20.6 | 35.8 | 22.3 | 34.7 | 26.7 | 19.3 |
| 20 - 49 | N | 147 | 14 | 8 | 7 | 13 | 116 |
|  | % | 10.2 | 12.8 | 7.8 | 14.3 | 12.9 | 9.7 |
| 50 - 99 | N | 151 | 9 | 10 | 5 | 9 | 129 |
|  | % | 10.5 | 8.3 | 9.7 | 10.2 | 8.9 | 10.8 |
| 100 - 149 | N | 113 | 5 | 11 | 4 | 6 | 97 |
|  | % | 7.9 | 4.6 | 10.7 | 8.2 | 5.9 | 8.1 |
| ≥ 150 | N | 269 | 10 | 14 | 6 | 14 | 234 |
|  | % | 18.7 | 9.2 | 13.6 | 12.2 | 13.9 | 19.6 |
| Chemotherapy regimens | |  |  |  |  |  |  |
| First cycle | N | 874 | 78 | 67 | 31 | 74 | 714 |
|  | % | 60.9 | 71.6 | 65.0 | 63.3 | 73.3 | 59.9 |
| Palliative  treatment | N | 676 | 27 | 38 | 21 | 42 | 589 |
|  | % | 47.1 | 24.8 | 36.9 | 42.9 | 41.6 | 49.4 |
| Chemotherapy classes | |  |  |  |  |  |  |
| Alkylating agent | N | 871 | 54 | 53 | 21 | 69 | 735 |
|  | % | 60.7 | 49.5 | 51.5 | 42.9 | 68.3 | 61.6 |
| Antimetabolite | N | 730 | 55 | 49 | 34 | 51 | 608 |
|  | % | 50.9 | 50.5 | 47.6 | 69.4 | 50.5 | 51.0 |
| Antitumor  antibiotic | N | 400 | 47 | 37 | 18 | 27 | 317 |
|  | % | 27.9 | 43.1 | 35.9 | 36.7 | 26.7 | 26.6 |
| Apoptotic  agent | N | 0 | 0 | 0 | 0 | 0 | 0 |
|  | % | 0.0 | 0.0 | 0.0 | 0.0 | 0.0 | 0.0 |
| Checkpoint  inhibitor | N | 1 | 0 | 0 | 0 | 0 | 1 |
|  | % | 0.1 | 0.0 | 0.0 | 0.0 | 0.0 | 0.1 |
| Conjugated  antibody | N | 3 | 0 | 0 | 0 | 0 | 3 |
|  | % | 0.2 | 0.0 | 0.0 | 0.0 | 0.0 | 0.3 |
| Corticosteroid | N | 77 | 13 | 3 | 5 | 4 | 62 |
|  | % | 5.4 | 11.9 | 2.9 | 10.2 | 4.0 | 5.2 |
| Enzyme | N | 18 | 3 | 1 | 1 | 0 | 13 |
|  | % | 1.3 | 2.8 | 1.0 | 2.0 | 0.0 | 1.1 |
| Microtubule  inhibitor | N | 474 | 42 | 36 | 11 | 40 | 386 |
|  | % | 33.0 | 38.5 | 35.0 | 22.5 | 39.6 | 32.4 |
| Monoclonal  antibody | N | 106 | 10 | 8 | 2 | 10 | 87 |
|  | % | 7.4 | 9.2 | 7.8 | 4.1 | 9.9 | 7.3 |
| m-TOR inhibitor | N | 2 | 1 | 0 | 0 | 0 | 2 |
|  | % | 0.1 | 0.9 | 0.0 | 0.0 | 0.0 | 0.2 |
| Proteasome  inhibitor | N | 24 | 3 | 4 | 0 | 0 | 20 |
|  | % | 1.7 | 2.8 | 3.9 | 0.0 | 0.0 | 1.7 |
| SUMOylation  inhibitor | N | 0 | 0 | 0 | 0 | 0 | 0 |
|  | % | 0.0 | 0.0 | 0.0 | 0.0 | 0.0 | 0.0 |
| Topoisomerase  inhibitor | N | 254 | 18 | 20 | 10 | 13 | 216 |
|  | % | 17.7 | 16.5 | 19.4 | 20.4 | 12.9 | 18.1 |
| Tyrosine kinase  inhibitor | N | 12 | 3 | 0 | 2 | 0 | 8 |
|  | % | 0.8 | 2.8 | 0.0 | 4.1 | 0.0 | 0.7 |
| Type of cancer | |  |  |  |  |  |  |
| Hematologic | N | 553 | 69 | 55 | 27 | 38 | 428 |
|  | % | 38.5 | 63.3 | 53.4 | 55.1 | 37.6 | 35.9 |
| Acute leukemia | N | 279 | 39 | 31 | 19 | 12 | 220 |
|  | % | 19.4 | 35.8 | 30.1 | 38.8 | 11.9 | 18.4 |
| Chronic  leukemia | N | 5 | 1 | 0 | 0 | 2 | 2 |
|  | % | 0.3 | 0.9 | 0.0 | 0.0 | 2.0 | 0.2 |
| Hodgkin's  lymphoma | N | 40 | 3 | 3 | 1 | 4 | 31 |
|  | % | 2.8 | 2.8 | 2.9 | 2.0 | 4.0 | 2.6 |
| Non-Hodgkin  lymphoma | N | 173 | 18 | 13 | 6 | 19 | 130 |
|  | % | 12.1 | 16.5 | 12.6 | 12.2 | 18.8 | 10.9 |
| Other | N | 56 | 8 | 8 | 1 | 1 | 45 |
|  | % | 3.9 | 7.3 | 7.8 | 2.0 | 1.0 | 3.8 |
| Solid tumor | N | 882 | 40 | 48 | 22 | 63 | 765 |
|  | % | 61.5 | 36.7 | 46.6 | 44.9 | 62.4 | 64.1 |
| Breast | N | 110 | 4 | 7 | 0 | 11 | 96 |
|  | % | 7.7 | 3.7 | 6.8 | 0.0 | 10.9 | 8.1 |
| Brain, spine,  and nerves | N | 6 | 0 | 0 | 0 | 1 | 5 |
|  | % | 0.4 | 0.0 | 0.0 | 0.0 | 1.0 | 0.4 |
| Female genital  organs | N | 69 | 3 | 3 | 2 | 2 | 62 |
|  | % | 4.8 | 2.8 | 2.9 | 4.1 | 2.0 | 5.2 |
| Gastrointestinal  tract | N | 300 | 11 | 14 | 8 | 23 | 256 |
|  | % | 20.9 | 10.1 | 13.6 | 16.3 | 22.8 | 21.5 |
| Head and neck | N | 43 | 2 | 2 | 1 | 1 | 39 |
|  | % | 3.0 | 1.8 | 1.9 | 2.0 | 1.0 | 3.3 |
| Liver or biliary  tract | N | 136 | 7 | 3 | 7 | 4 | 125 |
|  | % | 9.5 | 6.4 | 2.9 | 14.3 | 4.0 | 10.5 |
| Lung and  thorax | N | 42 | 2 | 6 | 1 | 4 | 36 |
|  | % | 2.9 | 1.8 | 5.8 | 2.0 | 4.0 | 3.0 |
| Male genital  organs | N | 16 | 0 | 2 | 0 | 2 | 14 |
|  | % | 1.1 | 0.0 | 1.9 | 0.0 | 2.0 | 1.2 |
| Prostate | N | 20 | 0 | 2 | 1 | 2 | 15 |
|  | % | 1.4 | 0.0 | 1.9 | 2.0 | 2.0 | 1.3 |
| Skin and bone | N | 39 | 1 | 3 | 1 | 1 | 33 |
|  | % | 2.7 | 0.9 | 2.9 | 2.0 | 1.0 | 2.8 |
| Urinary tract | N | 33 | 3 | 3 | 1 | 2 | 27 |
|  | % | 2.3 | 2.8 | 2.9 | 2.0 | 2.0 | 2.3 |
| Other | N | 68 | 7 | 3 | 0 | 10 | 57 |
|  | % | 4.7 | 6.4 | 2.9 | 0.0 | 9.9 | 4.8 |
| COVID-19 epidemic | N | 699 | 50 | 47 | 19 | 31 | 598 |
|  | % | 48.7 | 45.9 | 45.6 | 38.8 | 30.7 | 50.1 |
| Previous conditions | |  |  |  |  |  |  |
| CR-GNB infection | N | 24 | 16 | 0 | 0 | 1 | 12 |
|  | % | 1.7 | 14.7 | 0.0 | 0.0 | 1.0 | 1.0 |
| Methicillin-R  staphylococci infection | N | 30 | 3 | 9 | 2 | 3 | 17 |
|  | % | 2.1 | 2.75 | 8.7 | 4.1 | 3.0 | 1.4 |
| 3rd-gen cephalospoin resistant Enterobacterales infection | N | 6 | 0 | 0 | 3 | 0 | 4 |
|  | % | 0.4 | 0.0 | 0.0 | 6.1 | 0.00 | 0.34 |
| Fungal infection | N | 8 | 1 | 3 | 0 | 4 | 3 |
|  | % | 0.6 | 0.9 | 2.9 | 0.0 | 4.0 | 0.3 |
| Non-MDRO/  non-fungal  infection | N | 222 | 22 | 5 | 7 | 12 | 197 |
|  | % | 15.5 | 20.2 | 4.9 | 14.3 | 11.8 | 16.5 |
| Hospitalization  (days) | Median | 4 | 9 | 5 | 4 | 6 | 4 |
|  | IQR | 0-14 | 3-24 | 0-15 | 0-12 | 0-15 | 0-13 |
| Aminoglycoside  antibiotic (days) | Median | 0 | 0 | 0 | 0 | 0 | 0 |
|  | Max | 1 | 0 | 1 | 0 | 0 | 0 |
| Amphotericin  antifungal (days) | Median | 0 | 0 | 0 | 0 | 0 | 0 |
|  | Max | 10 | 0 | 7 | 0 | 0 | 10 |
| Anidulafungin  antifungal (days) | Median | 0 | 0 | 0 | 0 | 0 | 0 |
|  | Max | 14 | 0 | 0 | 0 | 8 | 14 |
| Azole antifungal  (days) | Median | 0 | 0 | 0 | 0 | 0 | 0 |
|  | Max | 36 | 24 | 30 | 17 | 7 | 36 |
| Carbapenem  antibiotic (days) | Median | 0 | 0 | 0 | 0 | 0 | 0 |
|  | Max | 81 | 17 | 39 | 23 | 27 | 81 |
| Cephalosporin  antibiotic (days) | Median | 0 | 0 | 0 | 0 | 0 | 0 |
|  | Max | 15 | 10 | 0 | 0 | 0 | 15 |
| Daptomycin  antibiotic (days) | Median | 0 | 0 | 0 | 0 | 0 | 0 |
|  | Max | 0 | 0 | 0 | 0 | 0 | 0 |
| Glycopeptide  antibiotic (days) | Median | 0 | 0 | 0 | 0 | 0 | 0 |
|  | Max | 28 | 18 | 14 | 27 | 20 | 28 |
| Linezolid  antibiotic (days) | Median | 0 | 0 | 0 | 0 | 0 | 0 |
|  | Max | 27 | 7 | 7 | 27 | 17 | 17 |
| Penicillin/BLI  antibiotic (days) | Median | 0 | 0 | 0 | 0 | 0 | 0 |
|  | Max | 26 | 26 | 16 | 22 | 18 | 26 |
| Polymyxin  antibiotic (days) | Median | 0 | 0 | 0 | 0 | 0 | 0 |
|  | Max | 17 | 10 | 7 | 14 | 17 | 8 |
| Sulfonamide  antibiotic (days) | Median | 0 | 0 | 0 | 0 | 0 | 0 |
|  | Max | 0 | 0 | 0 | 0 | 0 | 0 |
| Tigecycline  antibiotic (days) | Median | 0 | 0 | 0 | 0 | 0 | 0 |
|  | Max | 17 | 14 | 0 | 0 | 0 | 17 |
| 30-day mortality | N | 465 | 58 | 31 | 15 | 41 | 376 |
|  | % | 32.4 | 53.2 | 30.1 | 30.6 | 40.6 | 31.5 |
| CR-GNB, carbapenem resistant gram-negative bacteria; MRStaph, methicillin resistant staphylococci; 3GCRE, third-generation cephalosporin-resistant Enterobacterales; MDRO, multidrug-resistant organism. | | | | | | | |

| Supplementary Table S4 – Microorganism species (number and  proportion) from 1,435 bloodstream infections* | | |
| --- | --- | --- |
| Microorganism | Number | Proportion |
| *Escherichia coli* | 431 | 30.0% |
| *Klebsiella pneumoniae complex* | 254 | 17.7% |
| *Pseudomonas aeruginosa* | 147 | 10.2% |
| *Staphylococcus aureus* | 115 | 8.0% |
| *Enterococcus faecium* | 72 | 5.0% |
| *Enterobacter cloacae complex* | 60 | 4.2% |
| *Staphylococcus epidermidis* | 50 | 3.5% |
| *Acinetobacter baumannii complex* | 40 | 2.8% |
| *Streptococcus pneumoniae* | 37 | 2.6% |
| *Candida albicans* | 32 | 2.2% |
| *Bacteroides fragilis* | 31 | 2.2% |
| *Candida parapsilosis* | 21 | 1.5% |
| *Aeromonas spp* | 20 | 1.4% |
| *Enterococcus faecalis* | 19 | 1.3% |
| *Candida tropicalis* | 18 | 1.3% |
| *Streptococcus mitis / oralis* | 18 | 1.3% |
| *Stenotrophomonas maltophilia* | 15 | 1.0% |
| *Streptococcus grupo viridans* | 14 | 1.0% |
| *Serratia marcescens* | 11 | 0.8% |
| *Citrobacter freundii complex* | 10 | 0.7% |
| *Proteus mirabilis* | 9 | 0.6% |
| *Salmonella sp* | 9 | 0.6% |
| *Staphylococcus haemolyticus* | 9 | 0.6% |
| Other anaerobic | 50 | 3.5% |
| Other Streptococcus spp | 40 | 2.8% |
| Non-albicans candida | 20 | 1.4% |
| Other Enterobacterales | 20 | 1.4% |
| Other Gram-negative | 16 | 1.1% |
| Other non-fermentative gram negative | 15 | 1.0% |
| Other fungi | 12 | 0.8% |
| Other Enterococcus spp | 11 | 0.8% |
| Other coagulase negative staphylococcus | 10 | 0.7% |
| Other Gram-positive | 4 | 0.3% |
| *The sum of the numbers exceeds 1,435 due to polymicrobial infections. | | |

| Supplementary Table S5. Correlation matrix for infection types (phi coefficients), all episodes | | | | | |
| --- | --- | --- | --- | --- | --- |
|  | CRGNB | Methicillin-R staphylococci | 3rd-gen cephalosporin-R  Enterobacterales | Fungal | Non-MDRO/ non-fungal |
| CRGNB | 1.00 | -0.05 | -0.02 | -0.04 | -0.36 |
| MRGPC | -0.05 | 1.00 | -0.05 | 0.00 | -0.42 |
| 3GCRE | -0.02 | -0.05 | 1.00 | -0.04 | -0.28 |
| Fungus | -0.04 | 0.00 | -0.04 | 1.00 | -0.40 |
| Non-MDRO/ non-fungal | -0.36 | -0.42 | -0.28 | -0.40 | 1.00 |
| CRGNB, carbapenem resistant gram-negative bacteria; 3rd-gen, third generation; MDRO, multi-  drug resistant microorganism. | | | | | |

| Supplementary Table S6. Correlation matrix for infection types (phi coefficients), after restricting the dataset to the 114 episodes with more than one susceptibility profile group | | | | | |
| --- | --- | --- | --- | --- | --- |
|  | CRGNB | Methicillin-R staphylococci | 3rd-gen cephalosporin-R  Enterobacterales | Fungal | Non-MDRO/ non-fungal |
| CRGNB | 1.00 | -0.40 | -0.22 | -0.41 | -0.04 |
| MRGPC | -0.40 | 1.00 | -0.26 | -0.15 | -0.18 |
| 3GCRE | -0.22 | -0.26 | 1.00 | -0.23 | -0.12 |
| Fungus | -0.41 | -0.15 | -0.23 | 1.00 | -0.28 |
| Non-MDRO/ non-fungal | -0.04 | -0.18 | -0.12 | -0.28 | 1.00 |
| CRGNB, carbapenem resistant gram-negative bacteria; 3rd-gen, third generation; MDRO, multi-  drug resistant microorganism. | | | | | |

| Supplementary Table S7. Cross-validation results for the best (highest average AUC-ROC across infection types, weighted by the number of positive cases) parameterization of  each model. | | | | |
| --- | --- | --- | --- | --- |
| **Model** | **Parameterization** | **Chain length** | **AUC-ROC** | **Std dev** |
| RF | 100 trees with max. depth of 4 | 20 | 60.90% | 2.52% |
| MLP | 1 hidden layer with 2 neurons;  alpha = 0.1 | 120 | 59.96% | 2.89% |
| Logistic | L2 penalty with C=1 | 60 | 59.96% | 2.81% |
| GBM | 100 trees with max. depth of 1;  learning rate = 1 | 120 | 56.89% | 1.03% |
| AUC-ROC, Area Under the Curve of Receiver Operating Characteristic; Std dev, standard deviation. | | | | |

**Supplementary Material, Figures**

Figure S1. ROC curve for the best model (chain of 20 random forests of 100 trees with maximum depth of 4), evaluated on the test set for methicillin-R staphylococci infection.

Figure S2. ROC curve for the best model (chain of 20 random forests of 100 trees with maximum depth of 4), evaluated on the test set for 3rd-gen. cephalosporin-R Enterobacterales infection.

Figure S3. ROC curve for the best model (chain of 20 random forests of 100 trees with maximum depth of 4), evaluated on the test set for fungal infection.

Figure S4. ROC curve for the best model (chain of 20 random forests of 100 trees with maximum depth of 4), evaluated on the test set for non-MDRO/non-fungal infection (MDRO, multidrug-resistant organism).

Figure S5. Precision–recall curve for the best model (chain of 20 random forests of 100 trees with maximum depth of 4), evaluated on the test set for carbapenem-R Gram-negative bacilli infection.

Figure S6. Precision–recall curve for the best model (chain of 20 random forests of 100 trees with maximum depth of 4), evaluated on the test set for methicillin-R staphylococci infection.

Figure S7. Precision–recall curve for the best model (chain of 20 random forests of 100 trees with maximum depth of 4), evaluated on the test set for 3rd-gen. cephalosporin-R Enterobacterales infection.

Figure S8. Precision–recall curve for the best model (chain of 20 random forests of 100 trees with maximum depth of 4), evaluated on the test set for fungal infection.

Figure S9. Precision–recall curve for the best model (chain of 20 random forests of 100 trees with maximum depth of 4), evaluated on the test set for non-MDRO/non-fungal infection (MDRO, multidrug-resistant organism).

Figure S10. Shapley values for the best model (chain of 20 random forests of 100 trees with

maximum depth of 4), evaluated on the test set for methicillin-R staphylococci infection and computed using the SHAP package in Python.

Figure S11. Shapley values for the best model (chain of 20 random forests of 100 trees with

maximum depth of 4), evaluated on the test set for 3rd-gen. cephalosporin-R Enterobacterales infection and computed using the SHAP package in Python.

Figure S12. Shapley values for the best model (chain of 20 random forests of 100 trees with

maximum depth of 4), evaluated on the test set for fungal infection and computed using the SHAP package in Python.

Figure S13. Overall feature importance for the best model (chain of 20 random forests of 100 trees with maximum depth of 4): weighted average by positive cases for mean absolute Shapley values across infection types, derived from the test set and computed using the SHAP package in Python.

Figure S14. Mean absolute Shapley values for the best model (chain of 20 random forests of 100 trees with maximum depth of 4), evaluated on the test set for non-MDRO/non-fungal infection (MDRO, multidrug-resistant organism) and computed using the SHAP package in Python.
